# TGF-β in hematologic malignancies: molecular functions and clinical applications

**DOI:** 10.3389/fimmu.2026.1728730

**Published:** 2026-02-26

**Authors:** Ruiyang Li, Fang Wei, Mengbo Yang

**Affiliations:** 1 The First Clinical Medical College, Shanxi Medical University, Taiyuan, Shanxi, China; 2 Department of Hematology, The First Hospital of Shanxi Medical University, Taiyuan, Shanxi, China

**Keywords:** hematological tumors, targeted therapies, TGF-β, TGF-β signaling pathways, tumor microenvironment

## Abstract

Transforming growth factor-β (TGF-β) represents a family of multifunctional cytokines, primarily secreted by megakaryocytes, monocytes, T lymphocytes, bone marrow stromal cells, and other cell types. TGF-β plays an essential role in various physiological processes, including the regulation of cell proliferation, differentiation, apoptosis, and immune homeostasis. As a key immunoregulatory cytokine, TGF-β contributes to an immunosuppressive network within the microenvironment of hematologic malignancies by modulating the functions of both adaptive and innate immune cells. Current studies have shown that TGF-β is often highly expressed in major hematologic malignancies such as leukemia, lymphoma, and multiple myeloma (MM). It not only enhances immunosuppression by inhibiting effector T cell activation but also regulates tumor cell proliferation, apoptosis, and drug resistance. Meanwhile, strategies targeting the TGF-β signaling pathway have shown potential to improve immunotherapy responses in preclinical models of hematologic malignancies. Several such agents have now entered early-phase clinical trials, offering a promising direction for enhancing the efficacy of immunotherapy in these diseases. In this review, we outline the molecular mechanisms of TGF-β biosynthesis, activation, and signal transduction, discuss its functions across various immune cell types, and summarize recent progress and challenges in clinical research on TGF-β targeted therapies for hematologic disorders, with the aim of providing new perspectives for related treatment strategies.

## 1 Introduction

Transforming growth factor β (TGF-β) is a key pleiotropic cytokine that plays a critical role in a wide range of biological processes, including cell growth, differentiation, and immune responses. TGF-β can suppress or modulate the activation, maturation, and differentiation of both innate and adaptive immune cells, such as natural killer (NK) cells, dendritic cells (DCs), macrophages, neutrophils, and CD4^+^ and CD8^+^ T cells (1, 2). This immunosuppressive function creates favorable conditions for tumor growth and dissemination. Moreover, TGF-β also promotes tumor angiogenesis, supplying additional nutrients and oxygen to support tumor progression and metastasis. These diverse roles establish TGF-β as an important regulator in both normal physiology and various pathological states, especially in cancer. During tumor development, TGF-β influences cancer cells and the tumor microenvironment (TME) through autocrine and paracrine signaling mechanisms. The TME is a complex biological system composed of multiple cell types, extracellular matrix components, and signaling molecules (3, 4). In early tumorigenesis, TGF-β acts as a tumor suppressor by inhibiting cell proliferation and inducing apoptosis. However, as tumors advance, cancer cells often develop resistance to the growth−inhibitory effects of TGF-β and instead exploit its capacity to enhance metastasis and invasion (5, 6). This dual role lies at the heart of the complexity and therapeutic challenge in targeting TGF-β: specifically, how to inhibit its tumor−promoting functions without interfering with its tumor−suppressive activity in early−stage disease. Successfully navigating this challenge could allow us to retain TGF-β’s protective role during tumor initiation while blocking its pro−metastatic effects in advanced cancer, ultimately improving patient survival and quality of life.

Hematologic malignancies, including leukemia, lymphoma, and multiple myeloma (MM), are characterized by abnormal differentiation of hematopoietic cells and impaired immune surveillance. In recent years, despite significant progress in immunotherapy for solid tumors, its efficacy in hematologic malignancies often remains limited by the immunosuppressive properties of the TME (7). The TME comprises a complex ecosystem of tumor cells, immune cells, stromal components, extracellular matrix, and various signaling molecules. Through direct cell-cell interactions (such as the VCAM-1/VLA-4 and CXCL12/CXCR4 axes) and secretion of cytokines like SCF, IL-6, and IL-7, it finely regulates hematopoietic stem cell quiescence, proliferation, and differentiation. Notably, because hematopoietic tumor cells themselves derive from the immune system (e.g., T/B lymphocytes, myeloid cells, plasma cells), dysregulation of immune-related pathways can function as an intrinsic oncogenic driver. For example, TGF-β signaling is necessary for normal B cell differentiation, yet its functional inactivation in diffuse large B-cell lymphoma (DLBCL) may promote tumor proliferation. Conversely, in acute myeloid leukemia (AML), excessive TGF-β activity can block cellular differentiation (8). In addition, TGF-β secreted by tumor cells acts directly on immune effector cells in the TME: tumor-infiltrating CD8^+^ T cells and NK cells often show an exhausted or anergic phenotype, with high expression of inhibitory receptors such as PD-1 and TIM-3 (9). At the same time, immunosuppressive cell populations, including regulatory T cells (Tregs), myeloid-derived suppressor cells (MDSCs), and M2-type tumor-associated macrophages (TAMs), accumulate extensively within the microenvironment. These cells strongly suppress antitumor immune responses by secreting inhibitory cytokines (e.g., IL-10, TGF-β), depleting essential amino acids such as tryptophan, and generating reactive oxygen species (10, 11).

In hematologic tumors, TGF-β promotes tumorigenesis through distinct mechanisms depending on the malignancy type. In AML, high expression of TGF-β1 has been shown to support leukemogenesis by suppressing Th17 cell differentiation and impairing NK cell function, thereby helping leukemia cells evade immune surveillance (12). In chronic myeloid leukemia (CML), the BCR/ABL fusion protein upregulates TGF-β production, whereas the targeted agent imatinib controls disease progression in part by inhibiting TGF-β. In DLBCL, elevated TGF-β expression contributes to lymphoma growth by inducing regulatory T cell expansion (12–15). Together, these findings show that TGF−β acts through different mechanisms across hematologic malignancies, yet consistently influences tumor cell proliferation, immune evasion, and microenvironment remodeling. A deeper understanding of TGF−β’s structure, family members, and signaling pathways in hematologic malignancies can thus help clarify its molecular functions and provide a theoretical basis for clinical translation. In this review, we systematically summarize the mechanisms by which TGF−β contributes to the development and progression of hematologic malignancies, and discuss immunotherapeutic strategies targeting the TGF−β pathway along with recent research advances, aiming to inform further investigation and clinical application in this field.

## 2 TGF-β ligands and receptors

Ligands of the TGF-β signaling pathway are classified into two major subfamilies based on amino acid sequence homology and the signaling pathways they activate: the TGF-β/Nodal/Activin subfamily and the bone morphogenetic protein (BMP) subfamily. The TGF-β/Nodal/Activin subfamily includes multiple members, such as TGF-β1, TGF-β2, TGF-β3 (collectively referred to as TGF-β), Nodal, four activin isoforms, inhibin, growth differentiation factors GDF1, GDF3, GDF8, GDF9, and GDF15, as well as the Nodal coreceptor inhibitors LEFTY1 and LEFTY2 (16, 17). The BMP subfamily comprises 11 BMP members (BMP2–8a/8b/9/10), four additional GDFs (GDF5/6/7/10), and anti-Müllerian hormone (AMH) (18). The prototypical TGF-β subtypes (TGF-β1, β2, β3) and the inhibin β polypeptides that form activin and inhibin molecules contain nine characteristic cysteine residues. Eight of these form four intramolecular disulfide bonds, while the ninth participates in an intermolecular bond that links the two monomers. In contrast, inhibin α polypeptides, BMPs, and GDFs possess seven cysteines, with six forming intramolecular bridges and the seventh creating an intermolecular bridge (19, 20). Although ligands from both subfamilies share certain structural features, including a disulfide-linked dimeric architecture and a monomeric cysteine-knot fold, they differ significantly in their receptor-binding mechanisms and regulatory patterns. TGF-β/Activin ligands initially bind to type II receptors, which then recruit type I receptors to assemble a tetrameric receptor complex. Conversely, BMP ligands preferentially bind first to type I receptors before engaging type II receptors (21, 22).

TGF-β receptors are distributed between lipid rafts and non-raft domains on the plasma membrane (23). Ligand binding at the cell surface not only initiates downstream signaling but also triggers the internalization of both ligand and receptors. In particular, internalization of TGF-β receptors via clathrin−dependent endocytosis into EEA1−positive endosomes promotes signaling activation. This is attributed to the enrichment of Smad anchor for receptor activation (SARA), a Fab1, YOTB, Vac1 and EEA1 (FYVE) domain−containing protein, within EEA1−positive endosomes, which facilitates R−Smad activation (24–26). On hematopoietic cells, TGF-β ligands additionally bind to two coreceptors: the type III TGF−β receptor (TβRIII) and endoglin. TβRIII is a widely expressed coreceptor that binds all three TGF−β isoforms with high affinity. Its role is to concentrate ligand on the cell surface and promote engagement and signaling through TβRI and TβRII (27). However, TβRIII is not highly expressed in hematopoietic cells. Instead, endoglin – a coreceptor more specific to endothelial and hematopoietic cells – appears to be the predominant coreceptor in hematopoietic lineages. This is consistent with the observation that endoglin does not bind TGF−β2 and that hematopoietic cells show only weak proliferative inhibition in response to TGF−β2 (11, 28).

Both the membrane partitioning and internalization of TGF−β receptors are tightly regulated processes (29). A key regulator is Casitas B−lineage lymphoma (c−Cbl), a proto−oncogene encoding a ubiquitin E3 ligase that promotes TGF−β signaling by neddylating and stabilizing the type II receptor (TβRII). Knockout of c−Cbl reduces TβRII protein levels and desensitizes hematopoietic stem/progenitor cells to TGF−β, whereas c−Cbl overexpression stabilizes TβRII and sensitizes leukemia cells to TGF−β. c−Cbl conjugates neural precursor cell−expressed developmentally downregulated 8 (NEDD8), a ubiquitin−like protein, to TβRII at Lys556 and Lys567. This neddylation promotes TβRII endocytosis into EEA1−positive early endosomes while preventing its entry into caveolin−positive compartments, thereby inhibiting TβRII ubiquitination and degradation (30–32).

## 3 TGF-β signaling and biology

**Figure 1:**
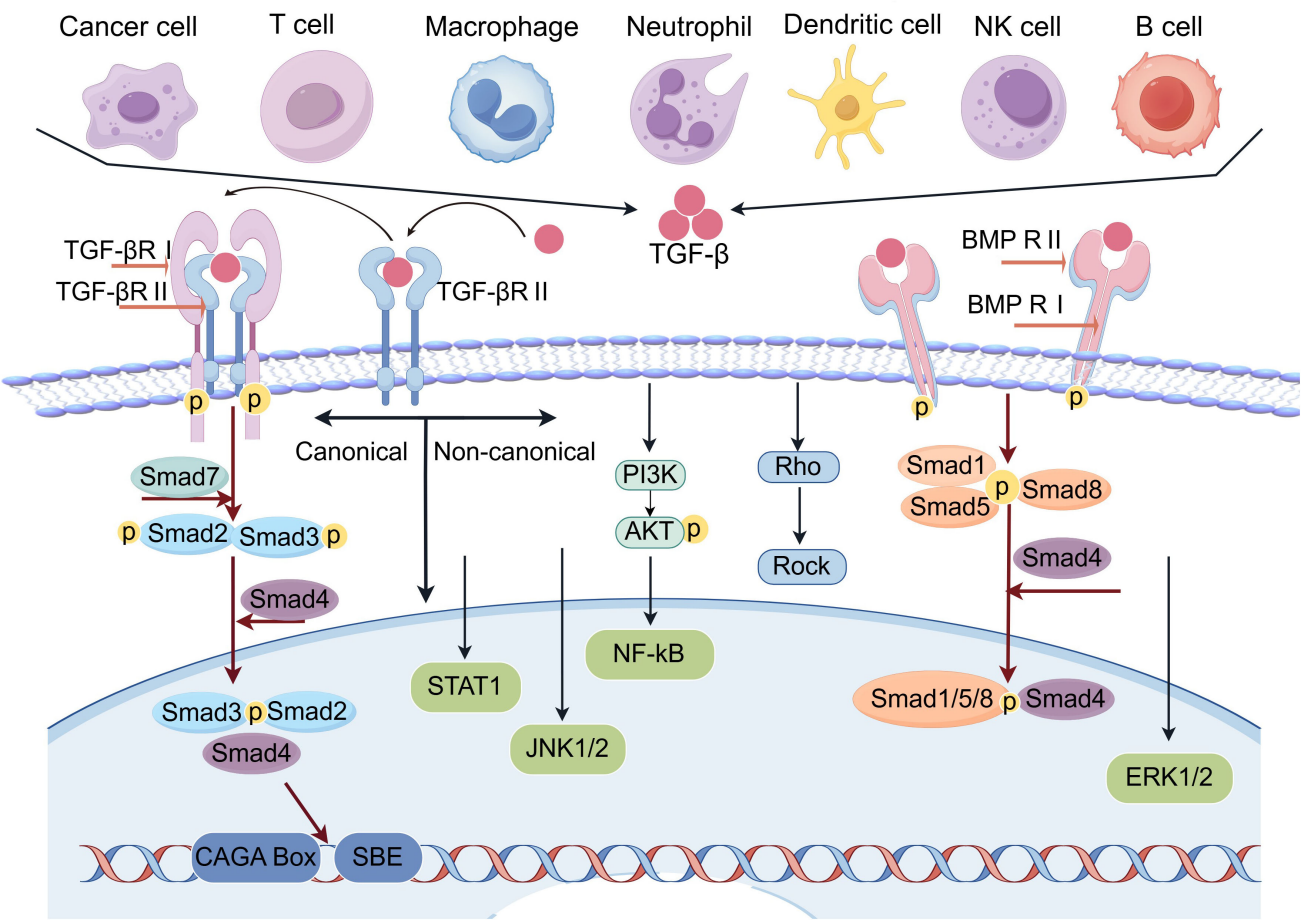
Canonical and non-canonical TGF-β signaling pathway (By Figdraw). In the TGF-β signaling system, the canonical Smad-dependent pathway is the core. Upon binding of TGF-β ligands to type II receptors (TβR-II), TβR-II recruits and phosphorylates type I receptors (TβR-I). Activated TβR-I subsequently phosphorylates receptor-regulated Smads (Smad2/3). Phosphorylated R-Smads form a complex with the common mediator Smad4, translocate into the nucleus, and bind to CAGA Box-containing promoters of target genes to regulate transcription. Non-canonical pathways include Rho, JNK1/2, NF-κB, ERK1/2, STAT1, and Smad1/5/8. In the Bone Morphogenetic Protein (BMP) signaling pathway, BMP ligands bind to type I/II serine/threonine kinase receptors, activating R-Smads (Smad1/5/8). These activated R-Smads then form a complex with Smad4 to regulate target gene transcription.

TGF−β signaling is initiated by the binding of TGF−β to its serine/threonine kinase receptors on the cell membrane, specifically the type II (TβRII) and type I (TβRI) receptors (33). Upon reaching target cells, TGF−β ligands first bind directly to TβRII, leading to the recruitment of TβRI. TβRII then trans−phosphorylates TβRI, activating its kinase domain and triggering downstream signaling cascades through cytoplasmic effector proteins (34, 35) (**Figure 1**). Each member of the TGF−β superfamily utilizes a specific combination of type I receptors, type II receptors, and receptor−regulated Smads (R−Smads). In canonical TGF−β signaling, TβRI and TβRII activate Smad2 and Smad3. In BMP signaling, ALK1, ALK2, ALK3, or ALK6, when paired with type II receptors such as BMPRII, ActRII, or ActRIIB, activate Smad1, Smad5, and Smad8 (36, 37). Hematologic malignancies often develop resistance to TGF−β by downregulating receptor expression or inhibiting the pathway through oncoproteins such as EVI−1 and Tax. In different leukemias, disease−specific oncoproteins disrupt signaling through distinct mechanisms. For example, PML−RARα interferes with assembly of the TGF−β receptor/SARA/Smad complex, preventing Smad2/3 phosphorylation. AML1−ETO, AML1−EVI−1, and EVI−1 suppress Smad3 DNA−binding and recruit the transcriptional corepressor CtBP (11). During the blast−crisis phase of CML, expression of EVI−1, normally low, becomes upregulated. EVI−1 binds to the MH2 domain of Smad3, inhibiting its DNA−binding and transcriptional activity. It further represses TGF−β target gene expression by recruiting the CtBP corepressor, thereby attenuating TGF−β signaling (38). Moreover, the AML1−EVI−1 fusion protein generated by the t (3, 21) translocation not only impairs the ability of AML1 to promote Smad3−mediated transcription, but also directly binds to Smad3 and antagonizes its transcriptional function (39, 40).

The cellular pathways regulated by TGF−β are categorized into two major types: the canonical Smad2/3/4 pathway and the non−Smad signaling pathways (33, 41). The canonical Smad2/3/4 pathway is activated when TGF−βRI phosphorylates Smad2 and Smad3. Following phosphorylation, these R−Smads associate with the common mediator Smad4 to form a heteromeric complex. This complex then translocates into the nucleus, where it regulates the transcription of target genes by interacting with various transcription factors, co−activators, and co−repressors, thereby directing cell−type−specific responses (33, 42). Smad3 can bind DNA directly by recognizing the Smad−binding element (SBE; 5′−GTCTAGAC−3′) and the CAGA motif (CAGA box) (43). In addition, TGF−β−activated Smad2/3, BMP−activated Smad1/5/8, and Smad4 can all bind to the GGC(GC)|(CG) sequence (44). Although Smad2 itself cannot bind DNA due to the absence of exon 3, it still exerts transcriptional effects by interacting with specific transcription factors such as POU domain class 5 transcription factor 1 (POU5F1), Nanog homeobox (NANOG), eomesodermin (EOMES), and transcriptional enhancer−associated domain (TEAD) proteins (45, 46). These five Smad proteins are classified as receptor−regulated Smads (R−Smads). As central signaling mediators, their expression levels and activities are tightly controlled through post−translational modifications, particularly within the linker region (47).

In addition to the canonical Smad2/3/4 pathway, TGF−β also activates multiple non−canonical pathways, such as those involving Rho GTPases, JNK1/2, NF−κB, ERK1/2, AKT, NFAT, p38, signal transducer and activator of transcription 1 (STAT1), and Smad1/5 (33). These non−canonical TGF−β pathways are essential for mediating a range of biological effects beyond conventional Smad signaling. They display dynamic and context−dependent properties: activation may occur rapidly upon TGF−β receptor engagement or be delayed until the induction of specific signaling mediators. Moreover, their activation can be either Smad−dependent or Smad−independent, and canonical and non−canonical pathways may cooperate or act separately to elicit cellular responses (48). TAK1 (MAP3K7), a serine/threonine kinase activated by TGF−β1, mediates several Smad−independent effects. It stimulates the MKK4−JNK and MKK3−p38 cascades to promote the expression of extracellular matrix components such as fibronectin and collagen I (49). TAK1 also engages in extensive crosstalk with the Smad pathway; it can upregulate the inhibitory Smad7 and bind to the MH2 domain of R−Smads to modulate their activity. In addition, downstream effectors of TAK1 (e.g., p38 MAPK) interact with Smads to fine−tune the expression of TGF−β target genes, thereby regulating processes including inflammation and apoptosis (50). These two pathways often function synergistically, forming positive feedback loops that enhance immunosuppression. For example, expression of the immune checkpoint protein VISTA is regulated by microenvironmental factors like TGF−β with involvement of the TAK1 pathway (51). In MM, TGF−β suppresses T cells, NK cells, and dendritic cells via the Smad3 pathway while concurrently regulating other immune processes through TAK1−related pathways. This combined action potently suppresses anti−myeloma immunity, promoting disease progression and drug resistance (52).

TGF-β signaling plays a key role in hematopoietic lineage commitment by modulating transcriptional programs in specific hematopoietic cell populations (53, 54). Studies indicate that activated Smad1 can colocalize with lineage−specific transcription factors at distinct genomic loci, such as GATA1 and GATA2 in erythroid progenitors, C/EBPα in myeloid progenitors, and PU.1 in lymphoid progenitors (8, 55). A model recently proposed by Challen and colleagues further shows that different hematopoietic stem/progenitor cell (HSPC) subtypes respond divergently to TGF−β. Specifically, TGF−β stimulates the proliferation of myeloid−biased hematopoietic stem cells (My−HSCs) while inhibiting the growth of lymphoid−biased hematopoietic stem cells (Ly−HSCs) (56). Another study found that the RNA−binding protein MUSASHI−2 is required for the proliferative response of HSPCs to low concentrations of TGF−β. Loss of MUSASHI−2 impairs HSPC quiescence, reduces My−HSC numbers, and decreases myeloid cell output *in vivo*. These changes are associated with significantly reduced levels of p57Kip2 and phosphorylated Smad2/3, suggesting that MUSASHI−2 participates in regulating the TGF−β signaling network (9, 57). Collectively, these findings support the view that excessive activation of inhibitory pathways such as TGF−β exacerbates the intrinsic hematopoietic failure in myelodysplastic syndromes.

## 4 Effects of TGF-β on immune cells

**Figure 2:**
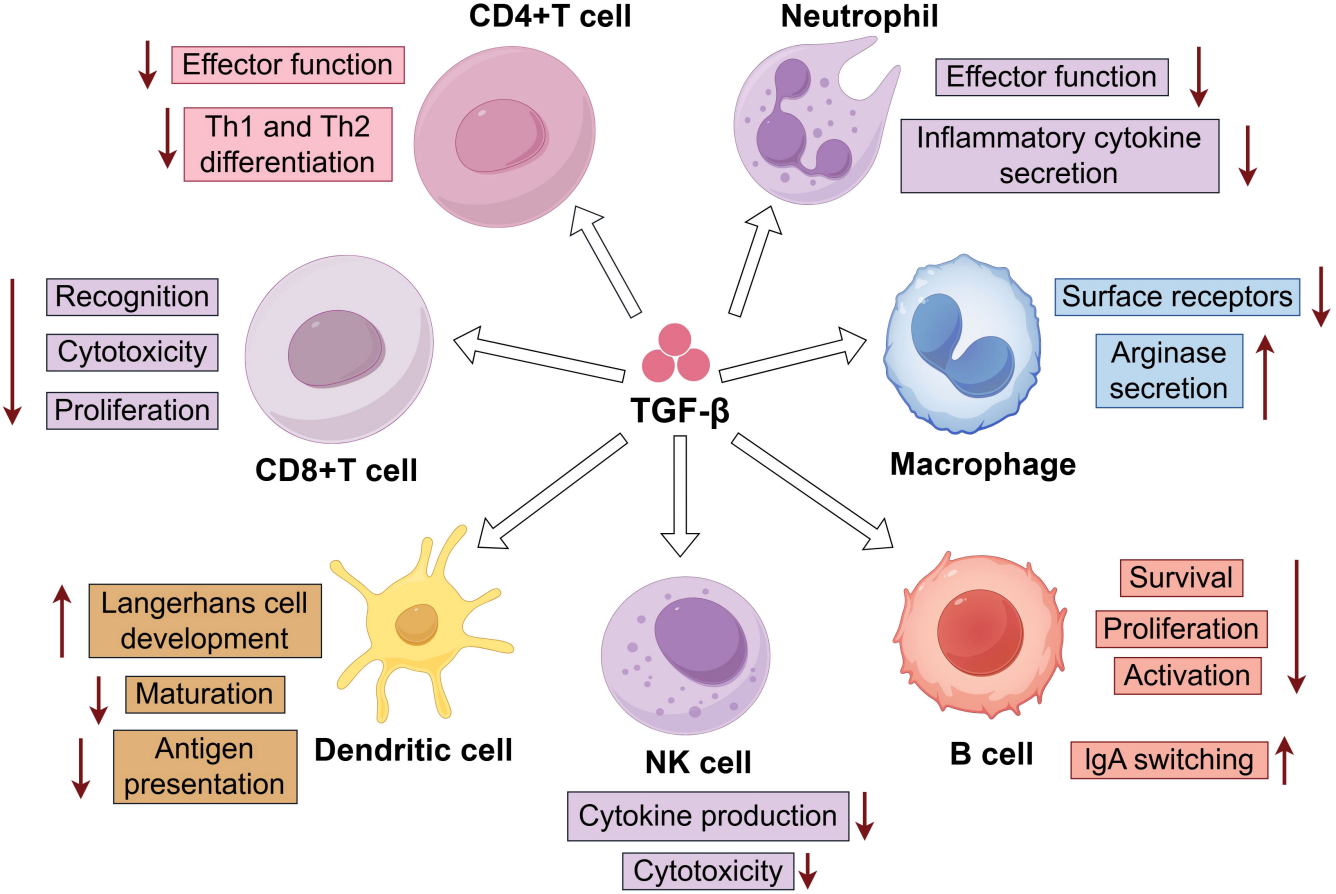
Regulation of Immune Cells by TGF-β (By Figdraw). TGF -β can regulate the effector function and Th1/Th2 differentiation of CD4+T cells; inhibit antigen recognition, cytotoxicity, and proliferation of CD8+T cells; downregulate the effector function and inflammatory cytokine secretion of neutrophils; reduce surface receptor expression and arginase secretion in macrophages; promote Langerhans cell development of dendritic cells while inhibiting their maturation and antigen presentation; decrease cytokine production and cytotoxicity of NK cells; and for B cells, inhibit their survival, proliferation, and activation while promoting IgA class switching.

Hematologic malignancies originate in the bone marrow, lymph nodes, and spleen, sites that are intrinsically involved in immune cell development and regulation. Stromal cells within these niches (such as osteoblasts, endothelial cells, adipocytes, and fibroblasts) physiologically express regulatory factors including TGF−β to maintain homeostasis. When tumor cells occupy these compartments, they exploit and amplify the inherent TGF−β signaling present in the microenvironment. For instance, in MM, myeloma cells interact with bone marrow mesenchymal stem cells, inducing them to secrete abnormally high levels of TGF−β. Even functional immune cells that enter the circulation from the thymus or bone marrow are rapidly suppressed by TGF−β (58, 59). Innate immune cells, including macrophages, dendritic cells, and natural killer (NK) cells, serve as the first line of defense against tumors. TGF−β primarily suppresses their antitumor activity and promotes immune tolerance. Adaptive immune cells, such as T cells and B cells, form the core of specific antitumor immunity. By controlling their differentiation, proliferation, and function, TGF−β helps establish a potent immunosuppressive microenvironment, which is closely linked to immunotherapy resistance in hematologic malignancies (60)(**Figure 2**).

### 4.1 T cells

The TGF-β signaling pathway plays a key role in determining T cell differentiation. Loss of TGF-β signaling disrupts the homeostasis of both CD4^+^ and CD8^+^ T cells. For instance, in mice whose CD4^+^ T cells express a single T−cell receptor, ablation of TGF−β signaling leads to a severe reduction in the peripheral T−cell pool (61, 62). In more pronounced cases, disruption of TGF−β−mediated control over CD8^+^ T−cell homeostasis can even promote cellular transformation, as demonstrated by the development of lymphoma in mice expressing a dominant−negative TGF−β receptor II (dnTGF−βRII) in T cells (63). In a mouse model where both CD4^+^ and CD8^+^ T cells express dnTβRII, strong antitumor immunity coincides with the expansion and heightened activity of tumor−specific CTLs (64, 65). Similarly, when tumor−specific CD8^+^ T cells are engineered to be resistant to TGF−β signaling via transduction with a comparable dnTGF−βRII construct prior to adoptive transfer, they efficiently infiltrate tumors, secrete cytokines such as IFN−γ, and clear tumor cells. These findings imply that dnTGF−βRII may exert a dominant effect independent of its canonical role in blocking TGF−β signaling, or that TGF−β regulates T−cell homeostasis in a dose−dependent manner (66). IIn AML patients, CD8^+^ T−cell exhaustion is especially marked, characterized by pronounced upregulation of inhibitory receptors such as PD−1, TIM−3, LAG−3, and CTLA−4, along with diminished secretion of effector cytokines including IL−2, TNF−α, and IFN−γ. This exhausted state involves the shift of memory T cells toward a terminally differentiated phenotype, leading to loss of long−term immune surveillance capacity. The functional impairment is not limited to CD8^+^ T cells but can also broadly affect other immune populations such as CD4^+^ T cells, thereby aggravating the overall immunodeficiency in the host. As a result, T−cell exhaustion represents not only a critical pathological feature of hematologic malignancies but also a key indicator for assessing disease severity and predicting clinical outcomes (67–69).

**Figure 3:**
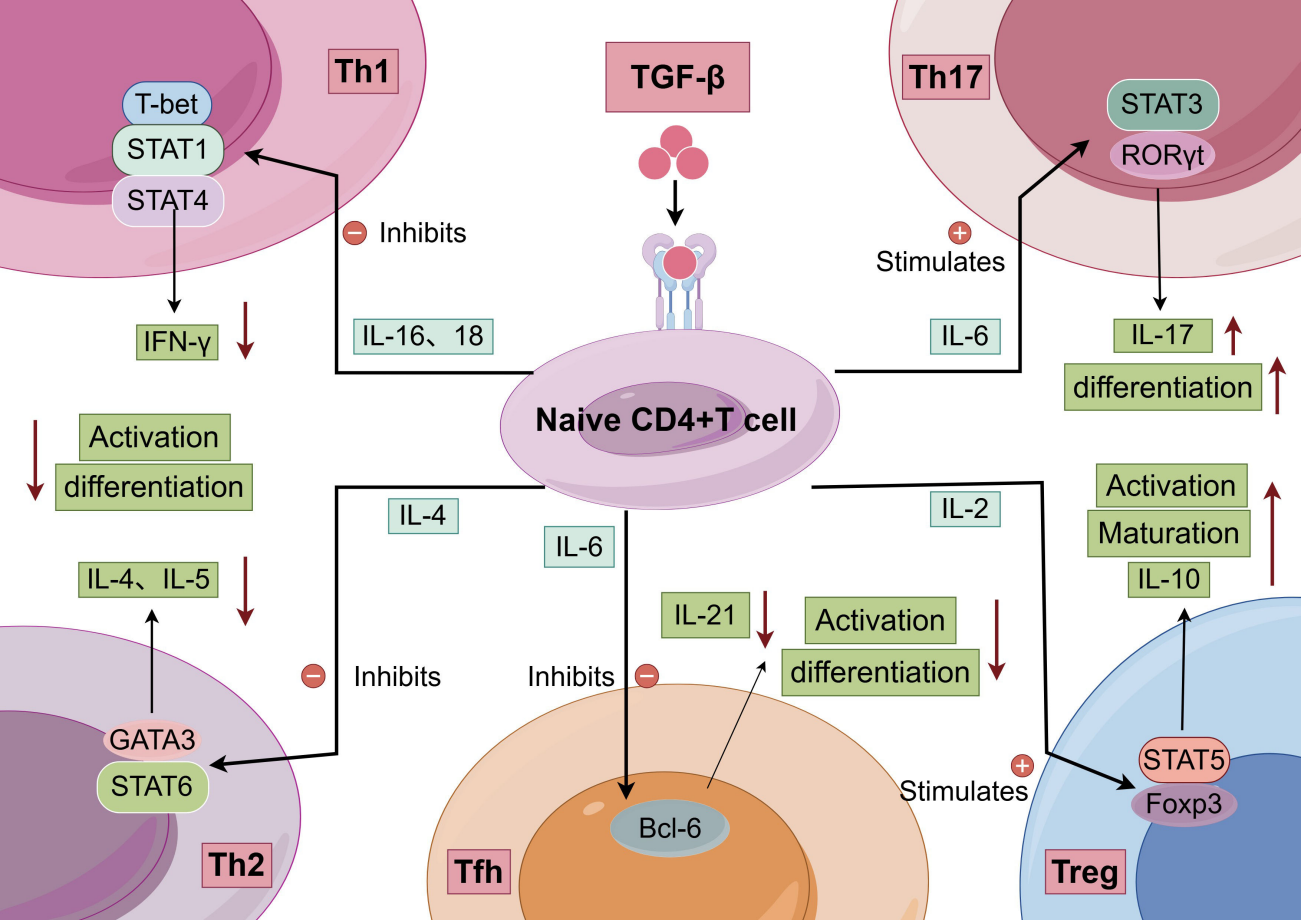
Regulatory network of naive CD4+ T cell differentiation into distinct helper T cell subsets (By Figdraw). Naive CD4+ T cells differentiate into Th1, Th2, Th17, Tfh, or Treg under the control of specific cytokines and transcription factors.

Tumor-derived TGF-β suppresses T helper 1 (Th1) responses by polarizing infiltrating T cells toward a Th2 phenotype (70), thereby promoting a less effective antitumor immune microenvironment. Concurrently, the TGF-β signaling pathway promotes Th17 cell development through synergistic interactions with the transcription factor RORγt (71). TGF-β also inhibits the differentiation of Th1 and Th2 cells, helping to maintain the dynamic balance among Th1, Th2, Th17, and regulatory T (Treg) cell subsets (**Figure 3**). For example, in tumor−bearing mice, exogenous IL−2 administration increases intratumoral Treg frequencies while reducing Th17 cell numbers (72). Conversely, antagonizing TGF-β activity via IL−7 supplementation effectively promotes Th17 differentiation (73). In MM, tumor cells produce high levels of TGF-β, which directly suppresses T−cell responsiveness and stimulates Treg proliferation, further amplifying immunosuppression. Importantly, TGF-β acts synergistically with cytokines such as IL−6 and IL−10 to establish a profoundly inhibitory tumor microenvironment, impairing the ability of T cells to sustain normal immune function (74, 75).

TGF-β is a central cytokine in the generation and function of regulatory T cells. It plays a critical role both in the differentiation of naïve T cells into induced Tregs (iTregs) and in maintaining the immune homeostasis of thymus−derived natural Tregs (nTregs). This regulatory function significantly shapes the immunosuppressive tumor microenvironment in hematologic malignancies (15, 76). *In vitro* studies confirm that activated naïve T cells can be efficiently converted into iTregs upon TGF-β stimulation, whereas differentiated T helper cells do not undergo this conversion under the same conditions. This difference likely reflects altered intracellular signaling in committed Th cells as well as reduced expression of TGF-βRI on the surface of activated T cells (77, 78). In MM, tumor and stromal cells secrete abundant TGF-β, creating a local cytokine gradient that favors iTreg generation. Clinical studies indicate that elevated TGF-β levels in patient bone marrow correlate positively with increased Treg frequency, and high Treg infiltration is associated with poorer responses to immunotherapies such as CAR−T cell therapy (58, 79).

### 4.2 Dendritic cells

The TGF−β signaling pathway significantly influences the function of dendritic cells (DCs). DCs are a heterogeneous group of bone marrow−derived immune cells with a strong capacity for antigen presentation. They play essential roles in initiating and shaping T−cell immune responses. Ex vivo studies show that DCs can take up foreign antigens and present them to naïve CD4^+^ T cells, leading to clonal expansion of effector T cells. Although DCs in cancer patients are often reduced in number and functionally impaired, potent DCs can be generated ex vivo from adherent peripheral blood mononuclear cells of cancer patients by culturing them with specific cytokines. Consequently, DCs engineered to present tumor antigens have the potential to induce robust antitumor immunity (80–82).

Some immunosuppressive factors, including TGF−β, IL−10, IL−6, and M−CSF, can impair dendritic cell differentiation and maturation. As a result, DCs in the tumor microenvironment often display an immature phenotype characterized by reduced responsiveness to activation signals and an inability to effectively activate T lymphocytes. Studies indicate that TGF−β1 does not affect expression of CD1α, a marker of immature DCs, but significantly downregulates CD83, a marker of mature DCs. These findings suggest that TGF−β1 does not interfere with DC differentiation but can inhibit their maturation (83). DCs lacking TGFBR2 fail to efficiently induce regulatory T cells, leading to excessive IFN−γ secretion and an imbalance between Th1 and Th17 cells. This dysregulation results in multi−organ inflammation and death in model systems (84). Furthermore, in environments with high TGF−β levels produced by tumor cells and TAMs, DC migration to tumor−draining lymph nodes is impaired because TGF−β suppresses expression of the chemokine receptor CCR7 on DCs (85, 86). Tumor−infiltrating DCs both secrete and respond to TGF−β in an autocrine or paracrine fashion, downregulating MHC class II molecules, the costimulatory molecules CD40, CD80, and CD86, as well as cytokines and chemokines such as TNF, IFN−α, IL−12, and CCL5 (87, 88).

TGF-β plays a central role in regulating the biology of Langerhans cells, specialized dendritic cells located in epithelial tissues that possess important immune and tolerogenic functions. Subsequent *in vitro* studies have shown that TGF−β can drive the differentiation of Langerhans cells from various human precursor cell types (89). Further support for the role of TGF-β comes from mouse models with conditional deletion of TGF−βRI or TGF−βRII, which confirmed that TGF−β signaling directly controls Langerhans cell development and maintenance (90). Moreover, mice with specific deletion of TGF-βRII or TGF-β1 in Langerhans cells display similar phenotypes, indicating that this pathway, particularly autocrine TGF-β signaling, is essential for Langerhans cells development or homeostasis (91).

### 4.3 NK cells

NK cells are innate lymphoid cells that play a key role in antitumor immunity by directly recognizing and killing tumor cells and by rapidly secreting chemokines and cytokines essential for this function (92). A study profiling functionally distinct NK−cell subsets in the bone marrow of MM−bearing mice found that KLRG1 (–) NK cells, which exhibit potent effector activity, rapidly and selectively decline in the bone marrow during disease progression. This decline closely correlates with reduced degranulation capacity of bone marrow NK cells *in vivo*. The abnormal shift in NK−cell subset distribution depends on imbalanced chemokine/chemokine−receptor axes in the myeloma microenvironment. Myeloma−driven upregulation of CXCR3 ligands together with downmodulation of CXCL12 acts as an exit signal, directing effector NK cells out of the bone marrow and thereby weakening antitumor immunity at the primary tumor site (93). NK cells primarily rely on receptors such as NKG2D, NKp30, and DNAM−1 to recognize tumors. TGF−β downregulates or impairs the expression of these receptors, diminishing the recognition of tumor−surface ligands, cytotoxicity, and overall immunosurveillance.

Beyond receptor modulation, the TGF−β/Smad signaling pathway regulates the production of IFN−γ and granzyme B by NK cells in response to antibody−dependent cellular cytotoxicity (ADCC). This regulation, which involves the transcription factor T−bet, impedes Th1 responses and can be counteracted by inflammatory signals (94). For example, NK−cell−derived IFN−γ is critical for stimulating the effector CD4^+^ Th1 cells needed to clear tumors; TGF−β attenuates both IFN−γ production and the lytic activity of NK cells (95). Exogenously administered TGF−β has been shown to inhibit NKp30 and NKG2D expression, reducing the ability of NK cells to kill target cells (96), The TGF−β pathway also influences murine NK−cell function and metabolism by inhibiting mTOR independently of the canonical Smad pathway. In human NK cells, however, the canonical TGF−β pathway appears to predominate (97). Furthermore, TGF−β can drive the conversion of NK cells into type I innate lymphoid cells (ILC1s), which show reduced immunosurveillance, upregulation of CTLA−4, and downregulation of IFN−γ. TGF−β can also promote a shift from type II to type III ILCs, which produce the immunosuppressive cytokine IL−17 (60). Recent studies using NK cells isolated from healthy donors demonstrate that platelet−derived TGF−β downmodulates NKG2D, leading to decreased IFN−γ production and impaired degranulation, functions essential for tumor killing (98). Together, these observations indicate that TGF−β exerts broad immunosuppressive effects on NK−cell cytotoxicity in cancer patients, suggesting that targeting this pathway could enhance NK−cell−mediated antitumor immunity.

### 4.4 Monocytes and macrophages

Early studies established that TGF-β primarily inhibits the proinflammatory response of macrophages activated by TLR ligands or cytokines. However, stimulation with TGF-β alone, in the absence of such signals, can promote the production of several inflammatory cytokines by myeloid cells (99). TGF-β also induces migration of human peripheral blood−derived monocytes and macrophages and enhances monocyte adhesion (100). It is suggested that TGF-β−recruited TAMs exhibit high phagocytic activity and can compete with dendritic cell (DC) function, thereby significantly impairing the ability of DCs to present tumor antigens to the adaptive immune system (101). TAMs acquire their characteristic phenotype by expressing high levels of TGF-β, IL-10, macrophage galactose N−acetyl−galactosamine−specific lectin 1 (MGL1), Dectin−1, CXCL10, CXCL9, and other interferon−responsive genes. Evidence also indicates that tumor−infiltrating MDSCs secrete elevated levels of TGF-β, which in an autocrine manner upregulates CD206, a marker associated with M2−like deactivation. This induced TGF-β may further promote alternative M2 macrophage activation by downregulating NF−κB expression (102).

In MM, the tumor microenvironment favors the polarization of macrophages toward the immunosuppressive M2 subtype. Multiple links exist between TGF-β and macrophages in the MM microenvironment. M2−like macrophages not only secrete TGF-β via integrin αVβ8 and MMP14 but also release TGF-β that indirectly activates fibroblasts (58, 103). IL−10 produced by MDSCs has also been reported to activate M2−like macrophages. Furthermore, TGF-β can recruit M2−like macrophages, which in turn secrete immunosuppressive factors such as IL-10 and TGF-β through the TGF-β/SMAD signaling pathway in concert with SNAIL (58). Additionally, the TGF-β/SMAD6/7 axis in macrophages suppresses anti−inflammatory responses alongside NF−κB. SMAD6 acts as an inhibitory factor by recruiting E3 ubiquitin ligases, leading to polyubiquitination of MYD88 and sequestration of adaptor protein spelling−1, thereby promoting inflammatory responses. This inhibitory mechanism is important for immune evasion, MM progression, and drug resistance (59). Moreover, TGF-β increases PD−L1 expression on M2−like macrophages, facilitating immune escape. It also upregulates CXCR4, which promotes monocyte migration to tumor sites by enhancing vascular permeability (52).

## 5 TGF-β-mediated crosstalk with other immune checkpoints in hematological malignancies

Immune escape in hematologic malignancies is often orchestrated by the activation of a broad immune checkpoint network. TGF−β transcriptionally upregulates multiple checkpoint molecules, such as PD−L1, IDO−1, Galectin−9, and VISTA, on tumor cells and immunosuppressive cells. This coordinated induction promotes T−cell exhaustion and represents a major obstacle to effective immunotherapy (104, 105). The interplay between TGF−β and PD−L1 is one of the best−characterized axes. TGF−β signaling directly promotes PD−L1 transcription via Smad3 binding to its promoter, establishing a positive correlation between TGF−β and PD−L1 expression across cellular components of the TME. This co−expression strongly suppresses tumor−infiltrating lymphocytes. In AML, overexpression of TGF−β1 can directly induce exhaustion of CD8^+^ T cells, characterized by marked upregulation of inhibitory receptors such as PD−1 and CTLA−4, alongside reduced secretion of key effector cytokines including IL−2, TNF−α, and IFN−γ. Furthermore, TGF−β enhances PD−L1 expression on antigen−presenting cells (APCs), thereby inhibiting T−cell activation and function via the PD−1/PD−L1 axis (106–108). The functional synergy between these pathways supports the rationale for dual−blockade strategies, such as PD−1/TGF−β bispecific agents. These approaches are currently under clinical investigation to overcome resistance to single−agent therapies and to enhance antitumor immunity (109–111).

IDO-1−mediated tryptophan depletion and kynurenine accumulation suppress T−cell function and shape an immunosuppressive microenvironment. In MM, IDO−1^+^ cells contribute to this milieu by promoting Treg differentiation and inhibiting cytotoxic T cells. Its expression is further amplified by stromal crosstalk and IL−6 signaling. Moreover, MM cells can upregulate IDO−1 and PD−L1 expression in stromal cells via the JAK–STAT1–NF−κB–IRF1 pathway (112). Similarly, TGF−β directly enhances Galectin−9 expression in AML blasts through Smad3. As a ligand for the T−cell surface checkpoint Tim−3, elevated Galectin−9 levels induce T−cell apoptosis or dysfunction (113, 114). In follicular lymphoma, Galectin−9 has been shown to impair rituximab efficacy, whereas blocking Galectin−9 can significantly restore antitumor immune responses (115). Its expression also correlates with reduced survival in chronic lymphocytic leukemia (CLL) and other cancers, underscoring its role in immune evasion and potential as a therapeutic target (116). VISTA, a B7 family member, promotes immune tolerance and tumor immune escape. Studies show that TGF−β−induced Smad3 activation increases VISTA expression in various cell types, including resting CD4^+^ T cells, primary human AML blasts, and chronic AML cells. Notably, TGF−β only induces VISTA in cells that already express detectable baseline levels; no induction occurs in cells lacking baseline expression, such as MCF−7 cells (117). In MM, VISTA frequently co−expresses with PD−1 and Tim−3, collectively driving T−cell exhaustion (51, 118). Although the precise mechanisms of TGF−β−mediated VISTA regulation in hematologic malignancies require further clarification, their interaction as key nodes in the immunosuppressive network remains a critical research focus.

## 6 Targeting TGF-β signaling for immunotherapy in hematologic malignancies

The immunoregulatory mechanisms of TGF−β in hematologic malignancies are complex and diverse. In leukemia, oncoproteins such as PML−RARα in acute promyelocytic leukemia (APL) block the assembly of the TβR−SARA−Smad complex, inhibiting Smad2/3 phosphorylation (15). In contrast, other fusion proteins like AML1/ETO and EVI−1 impair Smad3 DNA−binding by recruiting the transcriptional repressor CtBP (119). In MM, the role of TGF−β is multifaceted: myeloma cells promote their own survival and acquire drug resistance through autocrine and paracrine TGF−β signaling, while TGF−β also stimulates bone marrow stromal cells to secrete prosurvival factors such as IL−6, thereby enhancing tumor cell proliferation (120). Recent studies show that elevated TGF−β levels in the bone marrow of MM patients correlate with upregulated PD−1 expression on CD8^+^ T cells, directly impairing antitumor immunity. Moreover, TGF−β can promote bone marrow fibrosis and disrupt normal hematopoietic architecture, further accelerating disease progression (121). In lymphoma models, TGF−β acts mainly by regulating tumor−infiltrating immune cells. Animal studies indicate that TGF−β secreted by EL4 lymphoma cells reduces inflammatory factors (e.g., IL−2, GM−CSF) and effector T−cell infiltration in the tumor microenvironment (122). This “immune−desert” phenotype helps lymphoma cells evade immune surveillance and may explain why TGF−β−neutralizing antibodies alone show limited efficacy in some models (11). Studies suggest that selective inhibitors targeting TGF−β1 can avoid the unpredictable effects of pan−TGF−β blockade while enabling further investigation into the specific role of TGF−β1 in physiology and disease (123). In recent years, investigational agents targeting the TGF−β pathway have advanced significantly in hematologic malignancies. These agents can be grouped into three main classes based on mechanism: antibodies blocking TGF−β–receptor binding, TGF−β receptor fusion proteins, and small−molecule kinase inhibitors of TGF−β receptors (124). Several TGF−β signaling inhibitors, summarized in **Table 1**, are currently in various stages of development.

**Table 1:** TGF-β targeted therapies in hematologic malignancies.

Agent	Treatment	Target	Application	Experiment status	Clinical trial
Luspatercept	Monotherapy	TGF-β1/2/3	MDS	Phase III; Completed	NCT02631070
Galunisertib (LY2157299)	Small molecule kinase inhibitors of β receptors	TGF-βRI	Myelodysplastic syndromes	Phase I/II/III	NCT02008318
Vactosertib	Small-molecule inhibitors of TGF-β receptor	TGF-βRI	relapsed and refractory Myeloma	Phase I	NCT03143985
AVID200	Fusion proteins	TGF-β1/3	Myelofibrosis	Phase I	NCT03895112
STP705	Antisense nucleotides	TGF-β1/COX-2 specific siRNA	Hodgkin lymphoma	Phase I/II	NCT00368082/
TGF-β resistant LMP-specific T cells	Immune system activators	LMP+ TGF-β	relapsed Hodgkin lymphoma	Phase I	NCT00368082
BCMA-TGF-BETA CAR-T	Immune system activators	CD19 and BCMA+ TGF-β	relapsed and refractory Myeloma	Phase I	NCT05976555
SD-208	Small-molecule inhibitor	TGF-βRI kinase	MM	Preclinical	//

Many of these treatments have shown favorable tolerance and safety in addition to effectively suppressing TGF-β signaling in preclinical and clinical studies (125–128). Combining TGF-β−targeted therapies with other immunotherapies, such as PD−1/PD−L1 inhibitors or CAR−T cell therapy, can produce synergistic effects, thereby enhancing antitumor efficacy (4). For instance, in mouse models of CCK168 tumors, the combination of the anti−TGF−β monoclonal antibody NIS793 with the anti−PD−1 antibody spartalizumab improved the effectiveness of immune checkpoint inhibitors. This combination suppressed α−PD−1−induced Treg expansion, increased the CD4^+^ Th/Treg and CD8^+^ Th/Treg ratios, and reduced the population of myeloid MHC II^+^ antigen−presenting cells within responding tumors (129). Additionally, combining TGF−β inhibitors with chemotherapy or radiotherapy can help overcome treatment resistance and improve outcomes to some extent. Monoclonal antibodies work by specifically binding to TGF−β, thereby blocking its interaction with receptors. While such antibodies have demonstrated specific efficacy in certain solid tumors, their application in hematologic malignancies remains at an early stage. Furthermore, inhibition of TGF−β receptor signaling using the kinase inhibitor SD−208 suppressed the immunosuppressive effect of TGF−β on CD8^+^ and CD4^+^ ROR1−targeting chimeric antigen receptor (CAR) T cells (130).

AVID200 is a specific inhibitor targeting TGF−β1 and TGF−β3, currently under investigation primarily for myelofibrosis (MF) in hematologic malignancies. By selectively binding to and inhibiting the TGF−β1/3 signaling pathway, it reduces bone marrow fibrosis and collagen deposition while promoting recovery of normal hematopoietic function. The agent has completed phase I clinical trials (127). Its main advantage lies in the selective inhibition of TGF−β1/3, which avoids interference with the TGF−β2 pathway, potentially minimizing related side effects, and provides dual antifibrotic and pro−hematopoietic activities. However, AVID200 also presents certain limitations. Clinical studies indicate a relatively slow onset of action, with no significant improvement in bone marrow fibrosis observed within six months, suggesting that longer treatment may be needed for clear effects. Furthermore, excessive inhibition of TGF−β signaling could lead to over−activation of normal hematopoiesis, resulting in abnormally elevated blood cell counts. Currently, the drug’s indication is mainly focused on myelofibrosis, and clinical evidence supporting its use in other hematologic malignancies, such as acute leukemia or lymphoma, remains limited (127, 131).

Small−molecule inhibitors of the type I TGF−β receptor (ALK5) constitute the most well−established class of targeted agents in clinical development. Vactosertib (TEW−7197) is a highly selective TβRI kinase inhibitor that blocks downstream signaling by inhibiting Smad2/3 phosphorylation. In relapsed/refractory MM, its combination with pomalidomide has shown notable efficacy: it reduces bone marrow TGF−β levels, decreases the proportion of exhausted CD8^+^ T cells and enhances their cytotoxicity. This regimen achieved a 6−month progression−free survival rate of 82%, with clinical benefit observed in 75% of patients. However, the combination also carries safety risks. In the study, grade 4 neutropenia occurred in 20% of patients, requiring close monitoring and management. Additionally, grade 4 bilirubin elevation was seen in 5% of patients, and another 5% experienced grade 4 lipase elevation, underscoring the need to monitor potential hepatobiliary effects (126).

Galunisertib (LY2157299), developed by Eli Lilly and Company, is another TβRI kinase inhibitor. It acts by competitively blocking ATP binding to TGF−βRI, thereby inhibiting TGF−β−mediated phosphorylation and activation of Smad2 and Smad3. This agent has shown promising clinical activity in MM and myelodysplastic syndromes (MDS) (132). In patients with relapsed/refractory MM, Galunisertib−based combination therapy extended median progression−free survival (PFS) to 10–12 months, an improvement of approximately 3–4 months compared with conventional regimens. Among high−risk MDS patients, treatment resulted in a median PFS of 8–10 months. Furthermore, about 40–50% of intermediate− to high−risk MDS patients were able to reduce or discontinue red blood cell transfusions after therapy. The most frequent adverse events were hematologic toxicities consistent with bone marrow suppression: neutropenia (occurring in 60–70% of patients, with grade 3–4 in 25–30%), thrombocytopenia (50–60%, grade 3–4 in 20–25%), and anemia (40–50%, grade 3–4 in 15–20%). Peripheral neuropathy was observed in 15–25% of patients, primarily presenting as numbness, tingling, or paresthesia in the extremities. Other commonly reported adverse events, mostly grade 1 or 2, included fatigue (20%), diarrhea (17%), fever (12%), and vomiting (12%) (132, 133).

Luspatercept is an erythroid maturation agent that promotes late−stage erythropoiesis by binding to TGF−β superfamily ligands and inhibiting Smad2/3 signaling. Its pivotal phase III clinical trial (MEDALIST, NCT02631070) evaluated luspatercept in patients with lower−risk MDS with ring sideroblasts. The study enrolled 229 patients (125). Results showed that the primary endpoint, red−blood−cell transfusion independence for ≥8 weeks, was achieved in 37.9% of patients, significantly higher than the 13.2% rate in the placebo group. The key secondary endpoint (transfusion independence for ≥12 weeks) was reached in 43% of patients, compared with only 7.9% in the placebo group. Common adverse events included fatigue, headache, arthralgia, bone pain, hypertension, and diarrhea. Most events were mild to moderate but may affect quality of life in some patients. Furthermore, luspatercept efficacy appears more limited in patients without SF3B1 mutations, who tend to show suboptimal responses (125, 134).

## 7 Conclusions and future prospects

TGF-β plays a complex and critical role in the development and progression of hematologic malignancies. It influences tumor behavior by regulating cancer cell proliferation and apoptosis, and promotes tumor growth and metastasis through modulation of the tumor microenvironment. In recent years, significant progress has been made in therapeutic strategies targeting TGF−β. Various TGF−β inhibitors and cell−based immunotherapies directed against this pathway have shown promise in the treatment of hematologic tumors. Nevertheless, current research on TGF−β in these malignancies still faces several challenges. These include the complexity of TGF−β mechanisms across different disease subtypes, the need to further improve the safety and efficacy of targeted agents, and the lack of validated predictive biomarkers. In the future, as research continues to elucidate the role of TGF−β in hematologic cancers and as novel therapeutic technologies and agents are developed, TGF−β−targeted strategies are expected to assume an increasingly important role in clinical management, offering renewed hope for patients with hematologic malignancies.
